# Systematic Review: Outcomes by Duration of NPO Status prior to Colonoscopy

**DOI:** 10.1155/2017/3914942

**Published:** 2017-07-16

**Authors:** Aasma Shaukat, Ashish Malhotra, Nancy Greer, Roderick MacDonald, Joseph Wels, Timothy J. Wilt

**Affiliations:** ^1^Division of Gastroenterology, Veterans Affairs Medical Center, Minneapolis, MN, USA; ^2^Department of Medicine, University of Minnesota, Minneapolis, MN, USA; ^3^Center for Chronic Disease Outcomes Research, Veterans Affairs Medical Center, Minneapolis, MN, USA; ^4^Division of Anesthesiology, Veterans Affairs Medical Center, Minneapolis, MN, USA

## Abstract

**Background/Aims:**

Variation exists among anesthesia providers as to acceptable timing of NPO (“nothing by mouth”) for elective colonoscopy procedures. There is a need to balance optimal colonic preparation, patient convenience, and scheduling efficiency with anesthesia safety concerns. We reviewed the evidence for the relationship between NPO timing and aspiration incidence and colonoscopy rescheduling.

**Methods:**

We searched MEDLINE (1990–April 2015) for English language studies of any design and included them if at least one bowel preparation regimen was completed within 8 hours of colonoscopy. Study characteristics, patient characteristics, and outcomes were abstracted and verified by investigators. We determined risk of bias for each study and overall strength of evidence for primary and secondary outcomes.

**Results:**

We included 28 randomized controlled trials (RCTs), 2 controlled clinical trials, and 10 observational reports. Six studies reported on aspiration; none found that shorter NPO status prior to colonoscopy increased aspiration risk, though studies were not designed to assess this outcome (low strength of evidence). One RCT found fewer rescheduled procedures following split-dose preparation but NPO status was not well-documented (insufficient evidence).

**Conclusions:**

Aspiration incidence requiring hospitalization during colonoscopy with moderate or deep sedation is very low. No study found that shorter NPO status prior to colonoscopy increased aspiration risk. We did not find direct evidence of the effect of NPO status on colonoscopy rescheduling.

## 1. Introduction

Fourteen million colonoscopies are performed annually in the United States for screening, diagnosis, surveillance, and treatment of numerous colonic conditions. To optimize colon lining visualization, patients are advised to split the bowel preparation regimen such that half of the dose is taken in the evening prior to colonoscopy and the other half is taken ideally within 2–6 hours of the planned procedure [1–4]. In addition, some level of sedation (typically moderate or deep) is used in almost all colonoscopies to facilitate patient comfort and procedure quality [5, 6].

For both moderate and deep sedations, there is significant variation among anesthesia providers as to the acceptable timing of NPO (“nothing by mouth”) including how many hours prior to the planned procedure can the last bowel preparation dose be taken in order to minimize anesthesia risk (primarily aspiration). Practice guidelines from the American Society of Anesthesiologists Committee on Standards and Practice Parameters for preoperative fasting for healthy patients undergoing elective procedures suggest the following minimum fasting periods with the goal of minimizing anesthesia-related risks (primarily aspiration): 2 hours for clear liquids (e.g., water, fruit juice without pulp, carbonated beverages, clear tea, and black coffee), 6 hours for nonhuman milk, and 6 hours for a light meal (i.e., toast and clear liquids) [7]. The guideline authors note that the published clinical evidence is insufficient to clearly define a relationship between NPO status and risk of emesis/reflux or pulmonary aspiration. Furthermore, it is unclear how different bowel preparation agents would be classified (clear liquids or not), how the potential toxicity of bowel preparation agents might impact anesthesia-related risks, and how the volume of bowel preparation agent consumed might differ from the volume of liquids considered acceptable in the guidelines.

Several systematic reviews have reported the association of shorter time between preparation intakes with better quality of bowel preparation [1, 8, 9]. Based on these studies, recent gastrointestinal (GI) multisociety guidelines have recommended the use of split-dose bowel preparation for colonoscopy [4]. There is a need to balance optimal colonic preparation, patient convenience, and scheduling efficiency with safety concerns for an elective procedure. We reviewed the evidence on the relationship between timing of NPO and the incidence of aspiration and other anesthesia-related harms during elective colonoscopy.

## 2. Methods

This report is based on research conducted by the Evidence-based Synthesis Program (ESP) site at the Minneapolis VA Medical Center, Minneapolis, MN, funded by the Department of Veterans Affairs, Veterans Health Administration, Office of Research and Development, Quality Enhancement Research Initiative (QUERI).

Our primary outcomes were aspiration and rescheduled colonoscopies. Secondary outcomes of interest included diagnostic yield, completion rate, and adenoma detection rate. Other secondary and intermediate outcomes evaluated are discussed in our full report [10].

### 2.1. Data Source

We searched MEDLINE (OVID) for English language articles published from 1990 to April 2015. Detailed search strategy is presented in the Appendix. We also searched reference lists of guidelines, existing reviews, and included studies and received reference suggestions from stakeholders, technical expert panel members, and peer reviewers.

### 2.2. Study Selection

Abstracts of citations identified in the literature search were assessed for relevance, and full-text reports of studies identified as potentially eligible were obtained for an independent review by two investigators. We included studies of any design that reported outcomes following bowel preparation if at least one preparation was completed within 8 hours of the colonoscopy procedure. Only studies of adults, undergoing colonoscopy with moderate or deep sedation, in inpatient or outpatient settings, and reporting outcomes during colonoscopy or recovery from colonoscopy were included. We also identified population-based studies reporting aspiration during colonoscopy.

### 2.3. Data Extraction and Risk of Bias Assessment

Study characteristics (inclusion/exclusion criteria and preparation interventions or NPO status), patient characteristics, and outcomes were abstracted onto tables and verified by investigators. We also extracted information about timing of liquids other than bowel preparation agents allowed prior to colonoscopy from the 11 studies that reported that information.

Risk of bias (low, moderate, or high) was determined for each included study using a modification of the Cochrane approach [11]. Low risk of bias randomized controlled trials (RCTs) had adequate allocation sequence generation and allocation concealment, blinding, and few patients with incomplete data. Low risk of bias observational studies was prospective, enrolled consecutive patients, used appropriate methods for handling missing data (or no missing data), and characteristics of the NPO groups were similar.

We rated the overall strength of the body of evidence for our primary and secondary outcomes using the method reported by Owens et al. [12].

## 3. Results

**Figure 1:**
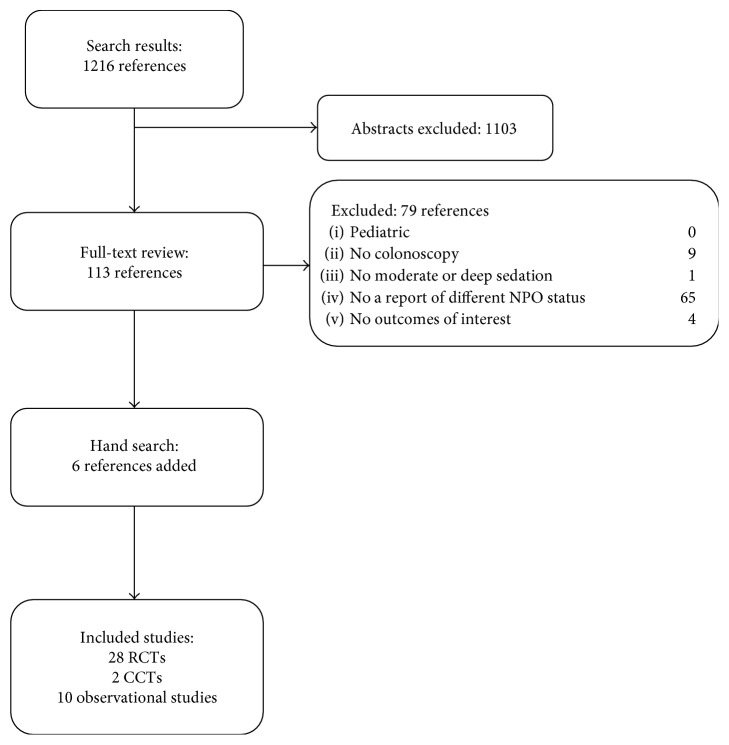
Literature flow chart.

Our literature search yielded 1216 abstracts or titles of which 40 were included (28 RCTs, 2 CCTs, and 10 observational studies), with a total of 22,936 patients [13–53]. The literature flow chart is seen in Figure 1. A summary of baseline characteristics for the 28 RCTs, 2 CCTs, and 10 observational studies is presented in Table 1. Detailed study characteristics and risk of bias criteria for all included studies are presented in the full evidence report [10].

**Figure 2:**
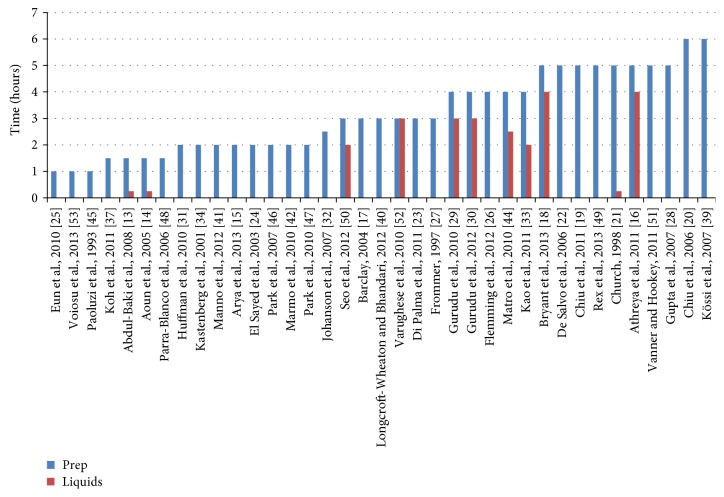
Minimum time from the end of bowel preparation to procedure (blue lines) or time before procedure when liquids were stopped (red lines). Three studies did not provide sufficient information to determine a minimum time from the end of preparation to procedure (Khan et al., 2010 [36], Kolts et al., 1993 [38], and Mathus-Vliegen and van der Vliet, 2013 [43]). Studies where patients were allowed liquids until time of procedure are indicated by a time of 0.25 hours. Citations are “author, year (reference number).”

Figure 2 displays minimum NPO times based on bowel preparation time and on time before the procedure that clear liquids were allowed. The majority of studies included an NPO time of 4 hours or less. The most frequently reported outcome (39 of the 40 included studies) was an intermediate outcome, quality of bowel preparation. Detailed findings for quality of bowel preparation are presented in the full evidence report [10]. We focus here on the 19 studies reporting our primary and secondary outcomes.

### 3.1. Incidence of Aspiration

Six studies (4 RCTs and 2 observational studies) reported on aspiration (Table 2). Sample sizes ranged from 115 to 1345 [29, 31, 41, 43, 44, 52]. In 5 of the studies, no aspirations occurred during colonoscopy [29, 31] or during colonoscopy or within the 30 days postcolonoscopy [43] or authors reported “no complications related to sedation” [41, 52]. In 4 of the studies, bowel preparation was completed at least 2 to 4 hours prior to colonoscopy; the fifth study used a split-dose regimen but did not report when the final dose was consumed with respect to colonoscopy time. In 2 studies, patients were allowed clear liquids up to 3 hours before the procedure.

One small RCT (outcome data for 115 of 125 patients randomized) reported one aspiration event requiring hospitalization during colonoscopy under moderate sedation [44]. The patient was described as severely obese (BMI = 40 kg/m^2^) but with no other obvious risk factors for aspiration. The patient was assigned to consume one liter of bowel preparation agent seven hours before colonoscopy and an additional one liter 4 hours before. Patients in this trial were allowed clear liquids until 2.5 hours before the procedure. We found low-strength evidence that shorter duration of NPO is not associated with a higher incidence rate of aspiration.

### 3.2. Additional Studies of Aspiration during Colonoscopy

Several hospital- or population-based studies reported on aspiration during colonoscopy. However, none documented duration of NPO status prior to the colonoscopy. In a large database study, the incidence of aspiration requiring hospitalization during 165,527 outpatient diagnostic colonoscopies in 100,359 Medicare patients age 66 years and older (mean age = 76 years) was 0.14% for patients having colonoscopy under deep sedation requiring anesthesia assistance (as identified by a CPT-4 code) and 0.10% for patients under moderate sedation without anesthesia assistance [54]. A study of 23,508 outpatient colonoscopies at 3 hospitals in Australia reported one case (0.004%) of aspiration requiring hospitalization in a patient undergoing colonoscopy with general anesthesia [55]. A study of 3155 colonoscopies performed with sedation managed by an anesthesiologist in adults at a single hospital in Italy reported that 0.16% of patients undergoing colonoscopy had an aspiration requiring “some intervention by an anesthesiologist” [56]. Aspirations requiring hospitalizations were not reported. Patients were instructed to fast according to guidelines in place at the time—clear liquids up to 2 hours before the procedure and a light meal (toast and clear liquid) up to 6 hours before the procedure.

### 3.3. Rescheduled Colonoscopies

One moderate risk of bias RCT (*n* = 113) reported rescheduled colonoscopies [38]. The percentage of rescheduled colonoscopies was significantly lower (*P* = 0.01) in the group that completed bowel preparation in the morning of the procedure (3%), taking a split-dose of a sodium phosphate regimen, than in groups consuming a polyethylene glycol solution (8%) or a castor oil solution (24%) in the evening before the procedure. Differences in the bowel preparation solutions between groups and imprecise reporting of timing of completion of bowel preparation limit our ability to draw firm conclusions about the role of NPO status on rescheduling. Strength of evidence was insufficient.

## 4. Discussion

We found low-strength evidence that risk of aspiration is not related to duration of NPO status prior to colonoscopy. However, few studies reported duration of NPO and aspiration or other risks related to colonoscopy. Studies were limited in size and were not population-based, limiting generalizability.

In hospital- and population-based studies, aspiration incidence requiring hospitalization during colonoscopy with moderate or deep sedation is very low (1 in 1000 or less) and on the order of magnitude commonly accepted for adverse effects of similar clinical importance due to other elective procedures. The largest study, and the only one conducted in the US, reported on patients age 66 and older (mean age 75 years). The applicability of results to younger individuals is uncertain though the reported percentage may overestimate aspiration risk. NPO status was not reported and the participants in these studies likely had wide ranges of timing from NPO to colonoscopy; many were likely longer than 2 to 4 hours. It is also important to acknowledge that in the US, there are no systematic tracking methods to track complications from colonoscopy—especially related to NPO status, and there is the possibility of under- or misreporting.

Many of the studies eligible for our review excluded patients with serious comorbidities. Few studies recorded mean or range of NPO status timing (including time of last ingestion of water, clear liquids, or bowel preparation substance). Furthermore, only 26 of 40 included studies reported on use of sedation during colonoscopy. Populations enrolled in eligible studies were broadly applicable to many individuals undergoing elective colonoscopy in the United States. Eligible studies typically included patients 45 to 65 years, and approximately 50% of patients were enrolled in studies done in the US. Nearly one-half of patients were male and two-thirds of colonoscopies were performed for cancer screening.

Other studies have focused on bowel preparation quality as their main outcome of interest and reported that shorter time from completion of colonic preparation to colonoscopy is associated with greater bowel preparation quality compared to longer time intervals. A recent systematic review assessed the efficacy of split- versus nonsplit-dose bowel preparation for quality of cleansing [9]. The authors included 29 studies and reported that an adequate preparation was obtained in 85% of patients in the split-dose group, compared to 63% in the nonsplit-dose group. Based on these studies, recent guidelines by the US multisociety task force on colorectal cancer [4] emphasized the importance of optimal bowel cleansing for a high-quality exam and recommended the use of split-dose bowel preparations, with the second half of the purgative given 4–6 hours before the time of colonoscopy. In addition, recent guidelines on quality benchmarks [57] have recommended that quality of bowel preparation should be monitored for colonoscopy and adequate bowel preparation should be achieved in ≥85% of colonoscopies. These reports make it essential for a colonoscopy program to monitor and maximize high quality of bowel preparation and balance the benefits of shorter NPO with any potential harms from risk of aspiration or other anesthesia-related complications.

Our findings indicate important knowledge gaps including the following: (1) an accurate assessment of aspiration requiring hospitalization and other serious anesthesia-related adverse events according to NPO status, (2) the extent of and reasons for variation in anesthesia NPO status practice and policy, (3) the effect of NPO status on procedure rescheduling and patient adherence and satisfaction, and (4) reasons for reduced patient adherence to recommendations for NPO status and bowel preparation. Future studies to close these knowledge gaps could improve care quality.

Specifically, to systematically assess duration of NPO status in relation to timing of colonoscopy and to record aspiration and other serious adverse events using standardized diagnostic criteria, prospective registries could be established. Providers would record timing of preparation, duration of NPO, and sedation procedures and then track adverse events over the next 48 to 72 hours. Future efforts could be directed towards developing standard methods to collate this information and initiate analyses to assess the association of duration of NPO and colonoscopy outcome. Special populations at higher risk of aspiration and other anesthesia-related outcomes would be of particular interest, such as elderly patients, patients with high comorbidities, and those with disabilities that limit ability to follow and complete the bowel preparation instructions. There is also a need to evaluate the effect of variable durations of NPO status prior to colonoscopy on patient satisfaction, adherence to colonoscopy, and endoscopy scheduling processes, including delays in timely receipt of colonoscopy. A better understanding of why some patients do not adhere to NPO status recommendations and methods to improve communication and adherence is needed. Alternative scheduling methods, including later but same-day colonoscopy, could also be evaluated to reduce “cancellations” due to NPO nonadherence.

Finally, evidence-based multisociety consensus guidelines that bring together patient representatives and members from anesthesia, gastroenterology, and general medicine are needed. Recommendations for NPO status also affect other gastroenterology procedures as well as procedures performed by other specialties (e.g., pulmonary and cardiology). Therefore, including representatives across a wide range of disciplines and procedures would be helpful in developing evidence-based recommendations targeted to specific procedures and likely benefits and harms. Important items in guideline development include determining the “clinically important” balance between critical outcomes to anesthesiologists, gastroenterologists (and other specialty groups performing procedures), and patients including aspiration rates due to NPO status, colonoscopy quality measures, resource use, and patient satisfaction and adherence.

In summary, aspiration incidence requiring hospitalization during colonoscopy with moderate or deep sedation is very low and on the order of magnitude commonly accepted for adverse effects of similar clinical importance due to other elective procedures. Participants in hospital- and population-based studies likely had wide ranges of timing from NPO to colonoscopy, and many were likely longer than 2 to 4 hours. No study documenting NPO status found that shorter NPO status prior to colonoscopy increased aspiration risk. We did not find direct evidence of the effect of NPO status on colonoscopy rescheduling.

## Figures and Tables

**Table 1:** Summary of baseline characteristics.

Characteristic	Mean (range) Unless otherwise noted	Number of studies reported
Total number of patients evaluated	22,936 (80 to 5175)	40
Randomized controlled trials, number of patients	9304 (80 to 895)	28
Controlled clinical trials, number of patients	740 (328 to 412)	2
Observational studies, number of patients	12,892 (100 to 5175)	10
Age of subjects, years (range of means)	57 (44 to 63)	34
Age of subjects, years (range of medians)	55 to 65	3
Gender, male, %	46 (28 to 81)	38
Indication for colonoscopy screening, %	61 (0^a^ to 100)	20
Location—USA/Canada, number of patients	12,208 (100 to 5175)	17
Location—Asia/Australia, number of patients	8045 (80 to 3079)	14
Location—Europe, number of patients	2683 (160 to 895)	9

^a^Two studies reported that screening was not an indication for colonoscopy. Chiu et al. [20] included participants who had colorectal neoplasms detected at a screening colonoscopy and were scheduled for a second colonoscopic examination for either elective polypectomy or endoscopic mucosectomy. Manno et al. [41] included participants with a positive fecal occult test or those in surveillance postpolypectomy.

**Table 2:** Summary of studies assessing aspiration events in relation to NPO status.

Author, year [reference number] Study design, sample size NPO status groups (1 = intervention; 2 = control)	Aspiration	
	NPO group 1	NPO group 2
*Gurudu et al., 2010* [29]	No episodes of bronchoaspiration were recorded, including in the procedures performed in patients taking same-day bowel preparation	
Observational: *n* = 1,345		
NPO status 1: ≥4 hours		
NPO status 2: >8 hours		

*Huffman et al., 2010* [31]	None of the patients in any group had clinical evidence of aspiration during their procedures	
Observational: *n* = 301		
NPO status 1: ≥2 hours		
NPO status 2: >8 hours		

*Manno et al., 2012* [41]	No major complications related to sedation	
RCT: *n* = 336		
NPO status 1: 2 hours		
NPO status 2: >8 hours		

*Mathus-Vliegen and van der Vliet, 2013* [43]	No events during 30-day period (from charts of patients and a complication database)	
RCT: *n* = 188 analyzed		
NPO status 1: hours unclear (split-dose exam (p.m.))		
NPO status 2: >8 hours		

*Matro et al. 2010* [44]	1.6% (1/61) were aspirated during the procedure	0/54
RCT, *n* = 115 analyzed		
NPO status 1: 4 hours (a.m. prep only)		
NPO status 2: 4 hours (p.m./a.m. prep)		

*Varughese et al., 2010* [52]	No sedation complications	
RCT: *n* = 136		
NPO status 1: ≥3 hours		
NPO status 2: >8 hours		

NPO: nil per os; RCT: randomized controlled trial.

## Appendix

### Search Strategy

Database: Ovid MEDLINE(R)

1. colonoscopy/
2. colonic.ti,ab.
3. (endoscop$ and (colon$ or rect$)).ti,ab.
4. or/1–3
5. cathartics/ or polyethylene glycols/ or phosphates/ or laxatives/ or senna extract/ or bisacodyl/ or cascara/ or enema/ or administration, oral/
6. (prepara$ or enema$ or cathart$ or (polyethylene adj glycol$) or phosphat$ or laxativ$ or (senna adj extract$) or bisacodyl or cascara or PEG or miralax or golytely or nulytely or halflytely or fleet or dulcolax or pico selax or bowel prep$ or bowel purgative or oral or liquid).mp.
7. 5 or 6
8. respiratory aspiration of gastric contents/ or respiratory aspiration/ or pneumonia, aspiration/ or dyspnea/ or vomiting/
9. (emesis or vomit$ or reflux or bronchoaspirat$ or aspirat$ or quality or detection).ti,ab.
10. 8 or 9
11. 4 and 7 and 10
12. limit 11 to yr=“1990 -Current”
13. limit 12 to English language
14. limit 13 to humans
15. limit 14 to (“all infant (birth to 23 months)” or “all child (0 to 18 years)” or “newborn infant (birth to 1 month)” or “infant (1 to 23 months)” or “preschool child (2 to 5 years)” or “child (6 to 12 years)” or “adolescent (13 to 18 years)”)
16. limit 14 to (“all adult (19 plus years)” or “young adult (19 to 24 years)” or “adult (19 to 44 years)” or “young adult and adult (19–24 and 19–44)” or “middle age (45 to 64 years)” or “middle aged (45 plus years)” or “all aged (65 and over)” or “aged (80 and over)”)
17. 14 not 15
18. 16 or 17
